# Management considerations for a critically ill 26‐gestational week patient with COVID‐19: A case report

**DOI:** 10.1002/ccr3.3886

**Published:** 2021-02-04

**Authors:** Mohamad Y. Khatib, Victor O. Olagundoye, Moustafa S. Elshafei, Fadi M. El Khatib, Ahmed S. Mohamed, Abdulqadir J. Nashwan

**Affiliations:** ^1^ Medical Intensive Care Department Hazm Mebaireek General Hospital (HMGH) Hamad Medical Corporation (HMC) Doha Qatar; ^2^ Obstetrics and Gynecology Department Women's Wellness and Research Center (WWRC) Hamad Medical Corporation (HMC) Doha Qatar

**Keywords:** COVID‐19, critical care, intubation, pregnancy, SARS‐CoV‐2

## Abstract

Pregnant women are potentially more susceptible to respiratory tract infections making them a high‐risk group. We describe the successful management of a 35‐year‐old pregnant woman, G3, P1, with a history of a cesarean section who tested positive for COVID‐19 at 26 weeks and required critical care support.

## Graphical abstract

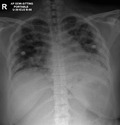

## 1 BACKGROUND

Pregnant women are potentially more susceptible to respiratory tract infections making them a high‐risk group. We describe the successful management of a 35‐year‐old pregnant woman, G3, P1, with a history of a cesarean section who tested positive for COVID‐19 at 26 weeks and required critical care support.

Coronavirus disease 2019 (COVID‐19) is a disease caused by severe acute respiratory syndrome coronavirus 2 (SARS‐CoV‐2) virus,^1^ which was declared by The World Health Organization as a pandemic on 11 March 2020. As of August 2020, a total of more than 21 million cases have been confirmed globally. Qatar recorded its first documented case of COVID‐19 on 29 February 2020 and has a total of 124 850 confirmed cases, with a total of 214 deaths (updated 28 September 2020).^2^ Pregnant women are potentially more vulnerable to respiratory tract infection including COVID‐19 3 compared with nonpregnant women due to anatomical, physiological, and immunological changes associated with pregnancy, putting them and their unborn babies at higher risks of adverse outcomes.^3^

## 2 CASE PRESENTATION

A healthy 35‐year‐old pregnant woman, gravida 3, para 1, with a history of previous caesarian section 3 years back for fetal distress was brought to the emergency department (ED) by emergency medical services (EMS) at 26 + 3 weeks’ gestation with a one‐day history of fever and shortness of breath. Three days earlier, she had attended the adult ambulatory care center (ACC) with a history of contact with COVID‐19, cough, and mild shortness of breath. On examination, she had apyrexia; her oxygen saturation was 99% and was systemically well. Nasopharyngeal swabs were taken for COVID‐19 RT‐PCR, and she was told to home quarantine while awaiting the results. Telephone consultation was to take place within the next two days but advised to return to the hospital if she experiences worsening symptoms.

**FIGURE 1:**
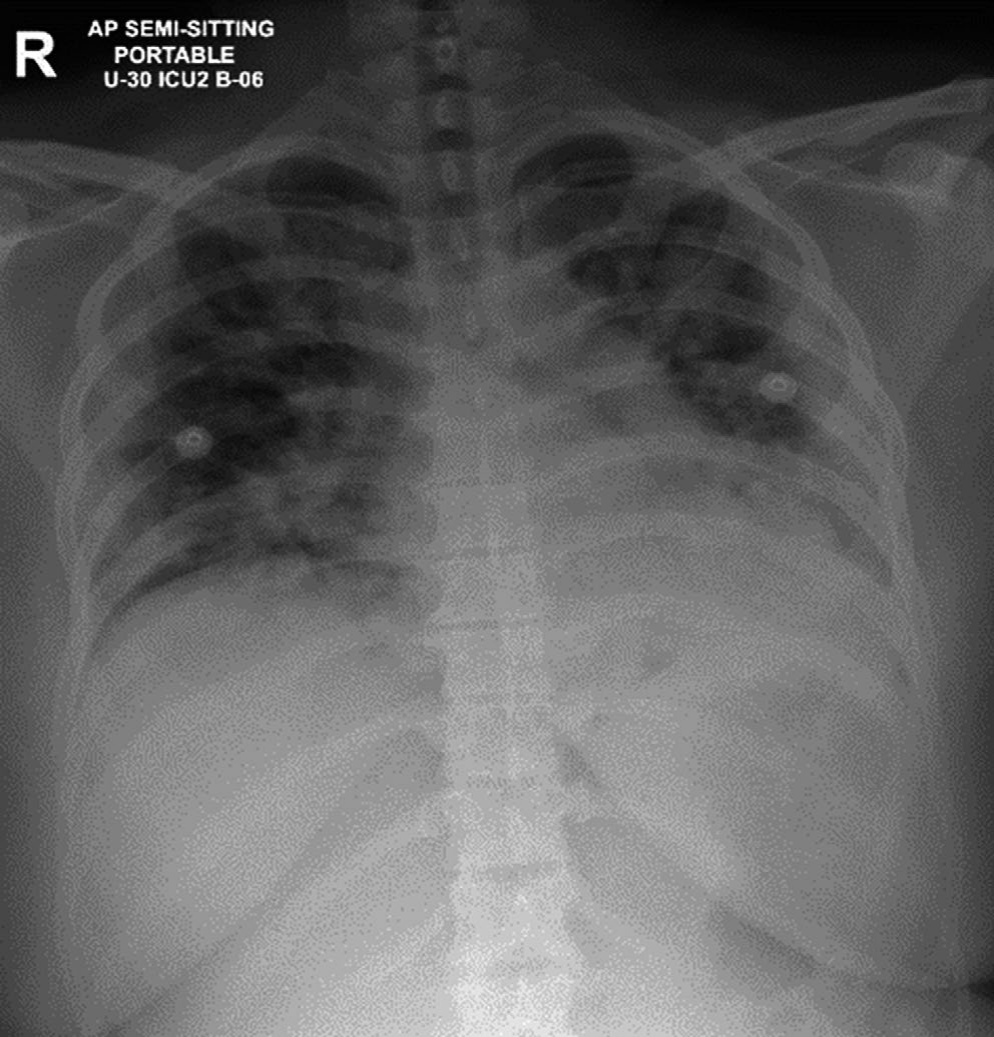
Chest X‐ray on admission

On admission to ED, she was tachypnea with a respiratory rate (RR) of 50 breaths/min (b/m), 88% oxygen saturation (SpO2) on room air, febrile at 38.5°C, and tachycardia with a heart rate (HR) of 120 beats/min. She was immediately transferred to the intensive care unit (ICU); chest X‐ray showed a typical COVID‐19 presentation (Figure 1).

Her investigations showed the following: hemoglobin 11.0gm/dl, WBC 9.2 × 10^3 uL, D‐dimer 0.39 mg/L FEU, fibrinogen 4.7 gm/L (NR 2.0‐4.1), LDH 334 U/L (NR 135‐214), myoglobin 80 ng/mL (NR 25‐58), CRP 70.2 (NR 0‐5), interleukin‐6 59 pg/mL (NR ≤ 7), and albumin level 21 (NR 35‐52). Echocardiogram was normal apart from minimal pericardial effusion. Her renal function and liver function tests were normal.

She was managed by a multidisciplinary team (MDT) involving the intensive care unit (ICU), obstetricians, and infectious disease teams. She was quickly put on continuous positive airway pressure (CPAP) high flow oxygen (Fio2) 50% which improved her oxygen saturation to 99%. She was started on piperacillin/tazobactam, azithromycin, immune globulin, convalescent plasma, dexamethasone 8 mg IV once daily, and enoxaparin 60 mg once daily increased to 100 mg BID postdelivery, along with other supportive measures. Bedside ultrasound showed an appropriately grown viable active fetus with a regular heart rate in cephalic presentation with the anterior upper placenta and mildly reduced liquor volume. After 30 minutes, her respiratory rate went up to 70 b/m (12‐20 b/m) and she expressed abdominal discomfort on the noninvasive ventilation (NIV); hence, it was discontinued and connected to a non‐rebreathing mask (NRBM) 15 L/min. Her tachypnea came down to 42 b/m on NRBM.

On day 2 of ICU admission, she was not tolerating the NRBM and continued to be tachypneic; hence, she was intubated and kept on mechanical ventilation. Following intubation, an MDT was held and a decision was made to deliver her by category 3 cesarean section (C/S) in the interest of fetal safety. The procedure was done without complication, and a baby boy was delivered in stable condition and transferred to the neonatal intensive care unit (NICU). The baby's COVID‐19 screening result was negative, and he remained in good condition at the time of writing.

On day 4 of ICU admission, the patient's oxygenation has improved, and trial extubation was carried out but failed as the patient became agitated. Her wound site was clean and healing well; postoperative hemoglobin was stable. There was minimal serosanguinous fluid in her drain which was removed.

**FIGURE 2:**
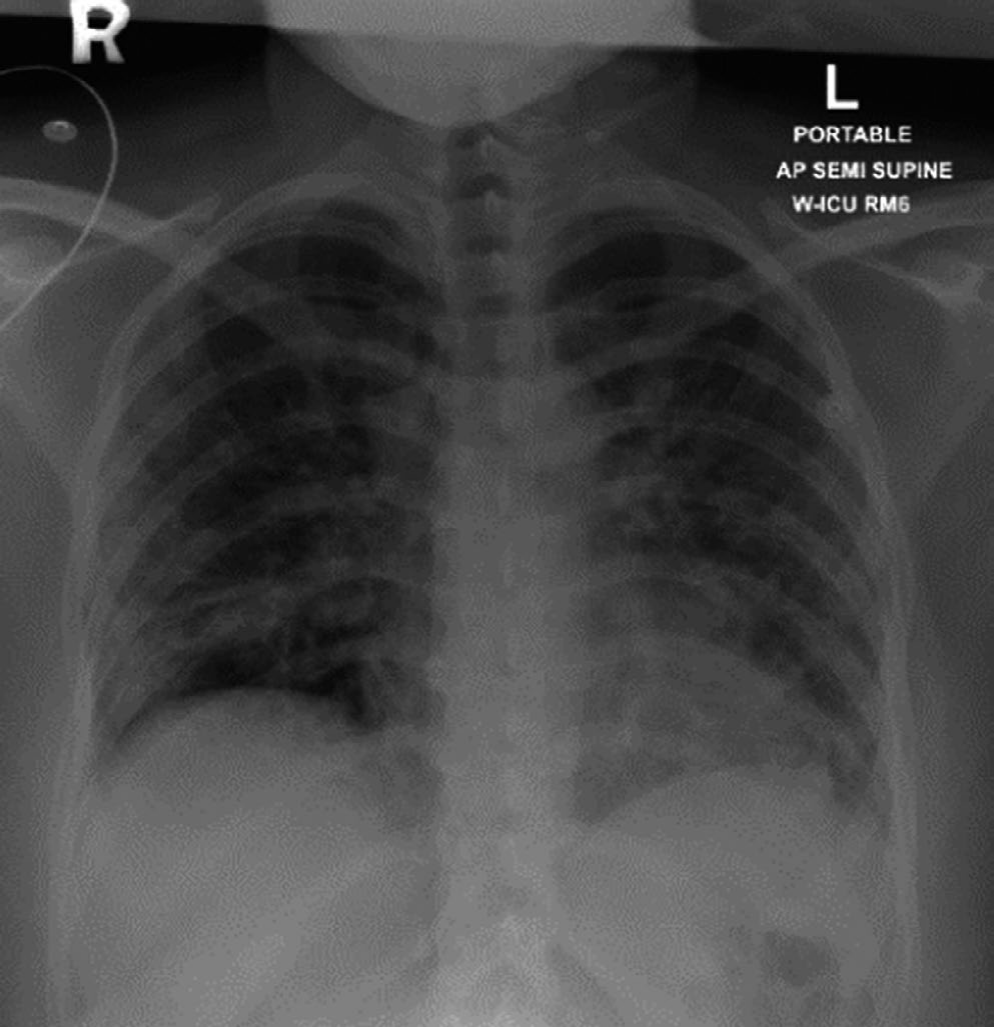
Chest X‐ray before discharge showing mild regression of the prior seen bilaterally airspace shadowing

She was successfully extubated on day 6 of hospital admission and kept on NRBM 10 L/M maintaining 98% oxygen. After extubation, she was kept on NRBM alternative with HFNC for one week maintaining 95%‐98% oxygen saturation. Then, oxygen requirement decreased and was kept on a nasal cannula. On day 11 of ICU admission, she has mobilized out of bed, and on day 12 of ICU admission, her oxygen saturation was within the normal range on the nasal cannula. Ivabradine 5 mg BID was added in view of tachycardia. She was discharged from ICU to home quarantine on day 13 of hospital admission. A chest X‐ray before discharge showed a mild regression of the previously seen bilateral airspace shadowing (Figure 2).

## 3 DISCUSSION

Pregnancy complicated with pneumonia is not uncommon and can account for up to 1.5 percent of hospital admission among pregnant women in the USA.^4^ As COVID‐19 is a novel virus, current knowledge on pregnant women who are severely or critically ill with the virus is still evolving and information is lacking on the overall impact on her unborn child especially in cases of severe prematurity (<28 weeks' gestation) which often presents a major management dilemma involving a delicate balance to ensure a good outcome for both mother and baby.

There are limited data on the course of COVID‐19 on pregnancy outcome, and the role played by normal immunogenic changes in pregnancy remains unclear. Some studies^5^, ^6^ have reported increased fetal and maternal morbidity and mortality, a higher rate of hospitalization and ITU admission among pregnant women compared with nonpregnant women, and possibly more severe disease in late pregnancy compared with early pregnancy, while other studies have reported no significant worsening of symptoms and possible shortening of clinical course in pregnant women admitted to hospital with severe or critical COVID‐19.^7^ A systematic review reported maternal mortality, stillbirth, and neonatal mortality rates of 1.6%, 1.4%, and 1.0% respectively.^8^ In another review of reports of 32 women with COVID‐19 during pregnancy, preterm delivery occurred in 47% of hospitalized women with one case of stillbirth and one case of neonatal death.^9^ Our patient was 26 + 3 weeks’ gestation at the time of admission. She was fit and well with no risk factor.

Pregnant women who are positive or in close contact with a confirmed or probable case of COVID‐19 must be closely monitored with strict instructions on what to do if there is a worsening of their symptoms. Our patient first presented to ACC with mild symptoms following contact with a patient with COVID‐19. She has no systemic symptoms, and examination was normal; therefore, she was advised home quarantine as per hospital protocol and to return to ED if her symptoms worsen.

There is no consensus on the best form of fetal monitoring or the optimal time for delivery in those who are severely or critically ill with COVID‐19. It is widely accepted that delivery should only be carried out for obstetric indications, and the mode of delivery should not be determined by the presence of COVID‐19. In one case report,^10^ fetal heart rate monitoring was carried out thrice daily for 20 minutes each time to detect any significant fetal abnormality.

Once our patient was intubated, the MDT made the decision for early delivery by category 3 C/S as soon as all arrangements including neonatal intensive care support were in place. It was felt that by the time a significant fetal heart rate abnormality is detected on intermittent fetal heart rate monitoring, it is highly possible the fetus might suffer irreversible hypoxic brain injury by the time all the necessary arrangements for emergency delivery can be put in place. Furthermore, our patient was managed in a dedicated COVID‐19 intensive care unit located more than 30 minutes' drive from the maternity and neonatal unit making it impossible to get the team across in a timely manner for category 1 C/S if there is a sudden maternal deterioration or acute fetal distress that poses an immediate threat to the life of the patient and/or her baby. The team felt that planned delivery in a calm environment would give the baby the best chance of survival and avoid the increased morbidity associated with an emergency cesarean section, especially if it happened in the middle of the night. We did not deliver her by category 1 C/S as there was no immediate threat to the life of the patient or her baby. The grade of the urgency of C/S depends on the presence or absence of fetal or maternal compromise.^11^ It is interesting to note that following delivery, there was a significant reduction in the patient's oxygen requirements.

## 4 CONCLUSION

Early aggressive management of pregnant women who are severely or critically ill with COVID‐19 by a multidisciplinary team is key to minimizing complications and early recovery. The timing of delivery should be determined by a multidisciplinary team approach on a case‐by‐case basis after a detailed discussion with the patient or her family. This should take into consideration both maternal and fetal clinical conditions and local circumstances to ensure desirable outcomes for both mother and baby.

## 5 COMPETING INTERESTS

The authors declare that they have no competing interests.

## AUTHORS' CONTRIBUTIONS

MYK, VOO, MSE, FME, ASM, AJN: collected data, involved in literature search, and prepared the manuscript. All authors read and approved the final manuscript.

## CONSENT FOR PUBLICATION

The consent for publication was obtained.

## ETHICS APPROVAL AND CONSENT TO PARTICIPATE

The article describes a case report. Therefore, no additional permission from our Ethics Committee was required.

## Data Availability

All data generated or analyzed during this study are included in this published article.
